# Behavioural pharmacology: 40+ years of progress, with a focus on glutamate receptors and cognition

**DOI:** 10.1016/j.tips.2006.01.009

**Published:** 2006-03

**Authors:** Trevor W. Robbins, Emily R. Murphy

**Affiliations:** Department of Experimental Psychology and University of Cambridge Behavioural and Clinical Neuroscience Institute, Downing Street, Cambridge CB2 3EB, UK

## Abstract

Behavioural pharmacology is an interdisciplinary field at the intersection of several research areas that ultimately lead to the development of drugs for clinical use and build understanding of how brain functions enable cognition and behaviour. In this article, the development of behavioural pharmacology in the UK is briefly surveyed, and the current status and success of the field is highlighted by the progress in our understanding of learning and memory that has resulted from discoveries in glutamate receptor pharmacology allied to theoretical and methodological advances in behavioural neuroscience. We describe the original breakthrough in terms of the role of NMDA receptors in hippocampal-mediated spatial learning and long-term potentiation, and review recent advances that demonstrate the involvement of glutamate receptor in working memory, recognition memory, stimulus–response learning and memory, and higher cognitive functions. We also discuss the unique functions of NMDA receptors and the fundamental role of AMPA receptors in processes that are common to some of these forms of memory, including encoding, consolidation and retrieval.

## Behavioural pharmacology and its origins

Behavioural pharmacology (or psychopharmacology) is at a practical interface between the discovery of a novel pharmacological agent that acts on the CNS and the assessment of its utility, whether clinically as a therapeutic agent or for analytical purposes in psychology and neuroscience. Consequently, it is important to use precise methods for assessing and interpreting the effects of drugs on behaviour in humans and experimental animals. Adequate preclinical screening of drugs by pharmaceutical companies is essential for selecting candidates for expensive Phase II and III clinical trials. Furthermore, appropriately measuring behaviour is vital to test speculative hypotheses about the consequences of neuronal plasticity based on the effects of drugs observed in cultured tissue slices or the consequences of genetic manipulations for a particular neurotransmitter receptor. Behavioural pharmacology must thus be an integral component of many neuroscience research programmes. In this article, we review briefly the origins of behavioural pharmacology in the UK and as a particular example of its success show how it has been instrumental in understanding more precisely the role of glutamate as a neurotransmitter in the processes of neuronal plasticity, with particular reference to learning and memory.

Behavioural pharmacology has flourished in the UK and worldwide, although it has evolved in several directions from different intellectual precursors and disciplines. The field derives from influences in pharmacology, psychiatry, neuroscience, ethology and several different traditions in psychology, each driven by a set of goals unique to the discipline. One of the main origins of behavioural pharmacology in the UK is work carried out in the Department of Pharmacology at University College London in the 1950s and 1960s. During this time, Roger Russell, the then Head of Department, encouraged Hannah Steinberg, in collaboration with Ian Stolerman and the late R. ‘Channi’ Kumar, to base their research programme on the behavioural effects of mixtures of stimulants and barbiturates in addition to opiates in experimental animals. Another major, but different, approach came from the field of psychiatry, where figures such as Max Hamilton and Alex Coppen had an interest in the treatment and aetiology of depression and other forms of mental illness. Hamilton constructed influential clinical scales for assessing both depression and anxiety in humans that are used today in evaluating the therapeutic efficacy of novel pharmacological agents. Both of these influences differed markedly from the development of behavioural pharmacology in the USA, which arose from a fusion of classic pharmacology and Skinnerian ‘operant’ psychology, as exemplified by the Harvard Medical School laboratory founded by Peter Dews, Roger Kelleher and Bill Morse. Although Harvard Medical School exerted an enormous influence over the methods used in the UK, in the late 1960s and early 1970s the late Jeffrey Gray at Oxford University and Susan Iversen at Cambridge University introduced two novel approaches to enrich behavioural pharmacology, the former depending on animal learning theory [1], and the latter depending on a fusion between neuropsychology, neurochemistry and traditional behavioural pharmacology [2]. Both approaches focused on the study of how drugs worked on defined neural systems, using powerful behavioural paradigms; these advances continue to influence the work of current researchers. Iversen's move to the Merck, Sharp and Dohme Neuroscience Centre at Terlings Park, Harlow, UK is just one example of how academic scientists moving to the pharmaceutical sector enabled the application of evolving psychopharmacological methodology to the evaluation of new compounds.

A landmark international CIBA symposium, ‘Animal Behaviour and Drug Action,’ held in London in 1963 and published a year later [3], contained several contributions from British psychopharmacology that reflected some of these origins. Reviewing the various contributions now, it is notable how contemporary many of the themes discussed at the meeting remain today. There were several chapters on the influence of drugs on memory (Jarvik) and learning (Bures and Russell), in addition to other current foci of research interest such as pharmacological interactions with frontal lobe functioning (Weiskrantz), amphetamine and neural reward mechanisms (Stein), and pharmacogenetics (Broadhurst). Furthermore, an entire section of the meeting was devoted to the relevance of the behavioural effects of drugs in animals and humans, thus presaging the current emphasis on translational neuroscience.

What has changed since then? Many of the fundamental conceptual questions have remained the same, although they have become much more refined as a result of major advances in theory and methodology in experimental psychology and major discoveries (e.g. new neurotransmitter systems and multiplicities of novel receptor mechanisms) in neuropharmacology. Although learning and memory were important topics in the CIBA symposium and behavioural pharmacologists had begun to work out how best to study these functions, a possible role for glutamate in learning and memory was largely unsuspected. However, Jeffrey Watkins and colleagues (Bristol, UK) were, at that time, investigating the possible role of glutamate as a neurotransmitter [4], which had been a controversial issue since Hayashi's initial observations [5] that central infusions of glutamate produced convulsions in experimental animals. In a recent review, Watkins [6] charts the stages by which glutamate gradually passed the stringent criteria for being a neurotransmitter substance, including structural and stereochemical specificity for actions at defined receptors, presynaptic release during axonal activity, the presence of an inactivation mechanism and the effect of specific antagonists on the production of excitatory postsynaptic potentials (EPSPs) by glutamate and the excitatory potential of the presumed neurotransmitter. Arguably the most impressive of all indications suggesting that glutamate is a neurotransmitter, however, came from behavioural pharmacology studies that demonstrated an intimate association between glutamate receptors and neuronal plasticity and complex behavioural functions, including learning and memory. These studies are reviewed here.

The anti-epileptic agent MK801 (dizocilpine), with a hitherto unknown mechanism of action, was first discovered to be a non-competitive antagonist at NMDA receptors by the UK Merck group at Terlings Park [7] and was one of the many tools that became available from pharmaceutical companies in the 1980s for the study of the role of glutamate receptors in learning. This topic has thus been a prime example of the crucial and sustained contributions of academic and industrial behavioural pharmacologists and neuroscientists in the UK in what is now an increasingly collaborative, international enterprise. In the search for new drugs, this research field is now being linked to the modelling of hitherto pathologically elusive clinical disorders, including Alzheimer's disease and schizophrenia, in addition to less-obvious examples such as general anxiety disorder and drug addiction.

## The behavioural pharmacology of learning and memory: focus on glutamate receptors

### Initial breakthrough

A key breakthrough in understanding the neural basis of learning has been the realization that there are different types of memory subserved by different neural systems. Thus, there is a declarative form of long-term memory for episodes and facts that appears to be mediated mainly by structures of the medial temporal lobe. Other forms of memory, such as perceptuo-motor skills and some forms of Pavlovian and instrumental learning (including stimulus–response habits) (see Glossary) appear to devolve to several structures such as the motor cortex, dorsal and ventral striatum, and cerebellum [8]. By contrast, information that is used for only a short time and has to be kept ‘online’ for that purpose requires ‘working memory’ processes that implicate the prefrontal cortex, certain areas of the posterior cortex and the hippocampus [9]. However, although these dissociable forms of memory imply different forms of information processing in different brain regions, they might nevertheless be mediated by similar cellular and molecular mechanisms. Thus, it is increasingly appreciated that neuronal plasticity processes represented by cellular phenomena such as long-term potentiation (LTP) [and also long-term depression (LTD)] might underlie different forms of learning and memory. The neuropharmacology of LTP itself is complex and beyond the scope of this article but it is known to depend crucially on glutamate-mediated transmission. Classically, blockade of the NMDA subtype of glutamate receptors blocks LTP in slice preparations, as originally observed by the British neuropharmacologist Graham Collingridge while working with Hugh McLennan and colleagues [10,11] However, the salient observation for behavioural pharmacology, discovered by Richard Morris and colleagues at Edinburgh, was that blockade of NMDA receptors also inhibited certain forms of learning, at comparable doses of the NMDA receptor antagonist to those that inhibited LTP. Thus, Morris and colleagues [12] showed that intracerebroventricular infusion of d-2-amino-5-phosphonopentanoic acid (AP-5), a competitive NMDA receptor antagonist, selectively impaired spatial learning in a water-maze escape task known to depend on the integrity of the hippocampus, but did not affect visual learning in the same maze. The remarkable observation was that this treatment did not have any effects on already established performance, indicating the effects were not obviously dependent on ancillary processes such as motivation, sensory perception or motor function. This selectivity of effect on task acquisition implies a direct action on associative learning mechanisms.

### The generality of the involvement of NMDA receptors in learning

The basic findings of the seminal study by Collingridge and McLennan have been substantiated by many subsequent demonstrations of impaired learning following manipulation of hippocampal NMDA receptors, whether by pharmacological experiments (generally employing rats) or genetic means (using mice). For example, Young and colleagues [13] showed that NMDA receptor blockade in the hippocampus impaired the normal learning of associations between an environmental setting or context and an electric shock (‘contextual fear’). Although it was initially thought that there might be a special relationship between NMDA receptors and spatial or contextual learning that is dependent on the hippocampus, other studies quickly showed that the effects of NMDA receptor blockade on learning were more general and implicated both other neural structures and other behavioural assays. For example, deficits in the acquisition of both fear conditioning and appetitive conditioning to discrete stimuli in rats (i.e. approach to food-predictive cues) were shown to depend on NMDA receptors within the amygdala [14,15]. A form of stimulus–response learning that is dependent on the dorsal striatum (approaching cues that are predictive of reward) has also been shown to depend on NMDA receptors [16], and Dalley and colleagues [17] recently showed that the consolidation of appetitive approach behaviour to a conditioned stimulus predictive of food delivery was similarly dependent on NMDA (in addition to dopamine D1) receptors in the ventral striatum. Furthermore, DiCiano and colleagues [18] showed that NMDA receptor antagonism in the core of the nucleus accumbens impaired acquisition of approach behaviour, whereas AMPA receptor antagonism impaired performance by disrupting discriminated approach. This growing catalogue of learning and memory deficits following NMDA receptor blockade has prompted several questions: what precise aspects of learning and memory are impaired, what is the role of other glutamate receptors (such as the AMPA subtype, which mediates fast depolarization by glutamate neurons, a prerequisite to NMDA receptor activation), and what therapeutic significance do these findings hold? For example, would it be feasible to enhance rather than impair learning or memory by manipulating glutamate-mediated transmission? New discoveries in behavioural pharmacology, enabled by the development of highly specific glutamatergic agents [19], are beginning to answer these questions.

### Pharmacological dissection of encoding, consolidation and retrieval memory processes

**Figure 1:**
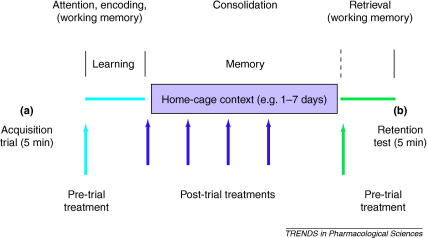
Stages of memory processing inferred from the results of neuropharmacological experiments. Depicted are two trials of any standard learning paradigm: **(a)** acquisition training and **(b)** retention testing. Pharmacological interventions before acquisition can potentially affect a range of sensory, perceptual, attentional, motivational and motor performance factors, in addition to learning and memory processes. To rule out the nonspecific factors that affect learning and memory *per se*, it is usually necessary to compare the effects on performance that is previously established (e.g. after a single trial of training as in the retention test). A lack of effect on retention indicates a specific effect on learning or memory-related processes such as encoding or memory consolidation. A lack of effect on initial training in acquisition accompanied by an effect on retention usually indicates a specific effect on memory consolidation or retrieval. The post-trial manipulations administered after the acquisition trial (e.g. in the home cage) cannot affect simple performance factors during the acquistion trial but do affect the hypothetical processes of memory consolidation. The time-limited nature of consolidation means that post-trial treatments soon after the first trial will affect memory consolidation, indicated by performance in the retention test, typically 1–7 days later. However, ineffective post-trial treatments at later time-points than immediate post-trial indicate the temporally limited nature of the consolidation process and rule out proactive effects of post-trial treatments themselves on the retention test. Working memory is an active process of memory that is usually engaged soon after perceptual processing to encode memory traces into passive storage. However, memories that are re-activated by memory cues also place retrieval memory traces into an active form in working memory for the guidance of behaviour (see [8,28] for further details).

The issue of which component of memory might be impaired concerns the processes implicated in initial encoding, later consolidation and storage, and the subsequent retrieval of the memory trace. These factors can be interrogated using so-called ‘one-trial’ memory tasks where (Figure 1) the agent of interest can be administered either before or just after initial learning and its effects on memory retrieval assessed by a subsequent retention test, possibly several days or more later. An agent that is only effective for a limited time-interval after training is said to affect consolidation rather than initial encoding of the trace. The agent can also be administered just before retention to test possible effects on memory retrieval (in addition to so-called ‘state-dependent’ effects, where the state change produced by the drug potentially acts as a memory-retrieval cue). Thus, the effects of blockade of NMDA receptors and other subtypes of glutamate receptors on different stages of memory can be determined. In one-trial ‘place memory’ tests, NMDA receptor blockade disrupted encoding and consolidation of the memory, but not retrieval [20]. Some of these effects were delay dependent, in the sense that they varied with the delay to the retention test, with greater deficits at long retention intervals [21]. A similar pattern of findings has been found for learned fear of contexts [22]. This implies that NMDA receptors have a selective role in the processes by which initially encoded events are made more permanent in long-term memory.

By contrast, blockade of AMPA and kainate receptors with a selective antagonist such as LY326325 (see Chemical names) disrupts not only encoding but also consolidation and retrieval of spatial memories, showing that fast synaptic transmission via AMPA receptors is required more generally for all of these processes [23]. This selectivity of the role of NMDA compared with AMPA receptors has subsequently been demonstrated for some other forms of memory mediated by the hippocampus, such as event-place associations in the rat, which are more readily linked to the forms of episodic memory deficits that are shown by patients with Alzheimer's disease. In this example, rats were trained to associate flavours with particular places where they had found those flavours in single episodes (or trials). Again, blockade of NMDA receptors within the hippocampus impaired encoding but not retrieval of the flavour–place associations, whereas blockade of AMPA receptors using CNQX (6-cyano-7-nitroquinoxaline-2,3-dione) disrupted encoding and retrieval [24]. Parallel studies using an ingenious test of memory consolidation based on non-spatial, socially transmitted food preferences also shows that NMDA receptors are not simply implicated in spatial encoding, but are also implicated in non-spatial aspects of ‘declarative’ learning [25].

### Recognition memory and working memory

**Figure 2:**
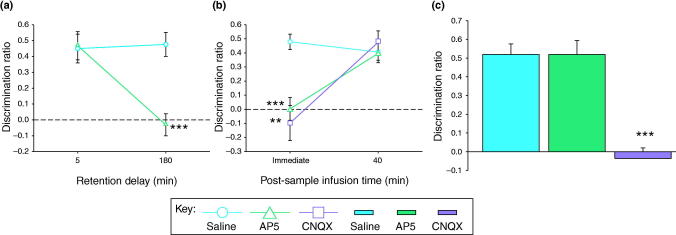
Results from a study on the effects of drugs on glutamate receptors following infusion of the drugs into the rat perirhinal cortex [28]. The study by Winters and Bussey [28] examined, for the first time, the effects of glutamate receptor manipulations on visual-recognition memory (memory for a familiar object) in experimental animals, following administration of the drugs at pre-acquisition, post-training trial or pre-retention test stages. This enabled identification of the nature of the involvement of glutamate receptors in the different phases of memory according to the logic described in Figure 1. Substances were infused directly into the rat perirhinal cortex via implanted cannulae because this region has been implicated in visual-recognition memory processes (see main text for further details). **(a)** NMDA receptor blockade in the perirhinal cortex following administration of AP5 impairs long-term but not short-term object recognition memory. **(b)** AMPA receptor blockade and NMDA receptor blockade in the perirhinal cortex following CNQX and AP5 administration, respectively, disrupts consolidation of object-recognition memory for up to 40 min post-trial. **(c)** AMPA receptor blockade but not NMDA receptor blockade in the perirhinal cortex disrupts retrieval in object-recognition memory during the retention test. ©2005 by the Society for Neuroscience [28].

Most recently, the involvement of NMDA and AMPA receptors has been demonstrated in another aspect of memory – the ability to recognize objects, which is markedly impaired in Alzheimer's disease. There is now strong evidence that object recognition is more dependent on a specific region of the medial temporal lobe, the perirhinal cortex, than on the hippocampus [26,27]. Recently, Winters and Bussey [28] have capitalized on this functional localization by administration of infusions of selective NMDA and AMPA receptor antagonists into the perirhinal cortex in rats at varying stages of performance of a visual object-recognition task. In this test, rats are allowed to interact with and explore a novel object before experiencing that now-familiar object again at a later time in conjunction with another object. Recognition memory can be inferred by the relative proportion of time the rats spend exploring the familiar and novel objects during a retention test carried out either a short or a longer time following the original presentation of the first object. The authors found that NMDA receptor blockade (using AP-5) impaired long- but not short-term object-recognition memory when infused before encoding the initial object (Figure 2a). This result suggests that NMDA receptors within the perirhinal cortex are not necessary for the initial perceptual encoding of the object but are required for the induction of synaptic plasticity required for the long-term storage of its trace. Moreover, NMDA receptor blockade soon after experience of the initial object also inhibited its subsequent recognition, showing that NMDA receptors are directly implicated in memory consolidation (Figure 2b). By contrast, NMDA receptor blockade immediately before the retention test had no effects on recognition memory, whereas AMPA receptor blockade (via CNQX) did disrupt retrieval, which parallels the findings for spatial memory in the hippocampus. AMPA receptor blockade also deleteriously affected all three stages of memory: encoding, consolidation and retrieval (Figure 2c). Thus, it appears that there is considerable generality in the glutamate mechanisms that mediate memory across not only domains (i.e. the nature of the memories), but also common memory processes, such as consolidation (Table 1).

Another way of defining the roles of glutamate receptor subtype function in memory is to employ mutant mice with targeted genetic deletions. Thus, evidence from mice that lack the AMPA receptor subunit A (GluRA/GluR1) supports the role of fast synaptic transmission in working memory functions, as measured in a delayed alternation paradigm where the mice simply have to remember which arm they last visited in a T maze [29]. The striking aspect of these data was that these mice were relatively unimpaired in a spatial task requiring longer-term memory and NMDA receptor involvement in the hippocampus, suggesting that different neuronal mechanisms within the hippocampus contribute to different (although probably interactive) types of information processing. One possible explanation for these possibly paradoxical results, given the normal close functional interdependence of NMDA and AMPA receptors, is that the targeted subunit deletion does not impair the function of the entire population of AMPA receptors such that NMDA receptor-mediated synaptic plasticity could be activated by a GluRA-independent mechanism. In previous studies, NMDA receptors had been implicated in spatial working memory functions in rodents, based on behavioural pharmacological evidence [21,30].

### Cognitive enhancement via glutamate receptors?

The explosion in knowledge about the nature of the glutamate receptors involved in learning and memory and the regulation of glutamate-mediated transmission begs the question of how such information might be used to develop new agents for improving memory and cognitive function [19]. The rather specific effects of NMDA receptor agents on the processes that link encoding to consolidation might encourage the further testing of this hypothesis by postulating selective enhancements with agonists. However, this strategy is weakened by the likelihood of serious side-effects (e.g. epilepsy and neurotoxicity) as a result of surplus activity of glutamate receptors. Some grounds for optimism are provided by effects reported on long-term memory recall in human volunteers by ‘AMPA-kine’ drugs that enhance excitatory glutamate transmission via their actions as positive allosteric modulators of the AMPA receptor [31]. Additionally, interest has focused on partial agonists that facilitate NMDA receptor activity in a more indirect way. For example, D-cycloserine (DCS) acts at the strychnine-insensitive glycine recognition site of the NMDA receptor complex [6], and, remarkably, has been shown to improve learning and memory in several animal tests, including visual-recognition memory in non-human primates [32] and maze learning and associative learning in both rats and mice [33,34]. Small beneficial effects have also been reported in clinical studies of schizophrenia [35] and Alzheimer's disease [36], although in some cases these promising results have proven difficult to replicate [37]. Notwithstanding this controversy, recent animal work has shown reproducible effects of DCS in experimental animals on a special sort of learning process, extinction learning, that is also dependent on NMDA receptors and has considerable implications for the treatment of other neuropsychiatric disorders.

The relatively recent discovery of another class of glutamate receptors, the metabotropic subtypes, has opened another strategic avenue in the modulation of glutamate-mediated transmission. These G-protein-coupled receptors (for reviews see [38,39]) modulate neuronal activity (versus the fast excitatory or inhibitory functions of ionotropic receptors such as AMPA and NMDA types) and are heterogeneously distributed, making them highly promising drug targets. Indeed, recent work has focused on their potential for improving cognitive deficits in schizophrenia [40], and applications are being investigated across the full spectrum of neurological and psychiatric disorders [41].

## The behavioural pharmacology of extinction learning: focus on glutamate receptors

Extinction occurs when the reinforcer (e.g. food or shock) associated with learning a particular task is omitted or withdrawn. Previously conditioned behaviour shows a gradual decline of responding during extinction, which suggested to early theorists that a form of ‘unlearning’ of the association must be occurring. However, recent findings have suggested that a new learning process occurs during extinction that has the effect of suppressing the conditioned stimulus–unconditioned stimulus (CS–US) association, although the memory of the association is preserved [42]. NMDA, but not AMPA, receptor antagonists have been known for some time to block extinction of learned fear, which means that an aversive CS continues to elicit fear in the absence of the US [43,44]. This retardation of extinction also occurs following intra-amygdaloid administration of AP-5 [44]. However, the striking recent finding has been that DCS accelerates extinction in the same learned-fear paradigm whether administered systemically or infused into the amygdala [45]. Several control experiments in this study showed that these effects could not be attributed to actions on fear-potentiated startle itself and that they were attenuated by HA966, an antagonist at the strychnine-insensitive glycine site [6].

Because current behavioural therapies for human anxiety disorders are explicitly based on the process of extinction (suppressing a learned association between a stimulus and anxiety by ‘relearning’ a new association that overrides the maladaptive state), the pharmacological enhancement of this process holds considerable promise for the treatment of these conditions. A recent study has directly tested this idea by showing that orally administered DCS produced a faster reduction of fear of heights than placebo in patients with anxiety, using a virtual-reality testing paradigm [46]. One of the problems with extinction therapy is that the effects learned in one situation fail to generalize to others, and thus its effects might be too limited to be of any therapeutic value. However, a recent study [47] has shown in rats that DCS not only facilitates extinction to an extinguished CS, but also reduces the fear of a second CS that does not undergo extinction itself: that is, the rats exhibited generalized fear extinction. Other forms of psychopathology such as drug addiction, which might arise in part from maladaptive conditioning processes, might also be treatable with this pharmacological strategy.

## The behavioural pharmacology of higher cognitive function: focus on glutamate receptors

The conceptualisation of extinction as a special form of inhibitory conditioning, and its apparent dependence on prefrontal cortical mechanisms [48], is consistent with the hypothesis that glutamate receptors are implicated in forms of plasticity that are important for the cognitive control (often termed ‘executive’) functions of the prefrontal cortex. These cognitive functions, however, operate rapidly over short time-scales and endow the animal with the flexibility to respond to a changing environment. One such function is working memory, which requires the maintenance of information ‘online’ over short periods to guide future behaviour while resisting distractions [9]. Working memory functions that are dependent on the prefrontal cortex (PFC) are impaired by NMDA receptor antagonists such as ketamine (a non-competitive NMDA receptor antagonist) in humans [49]. A complementary prefrontal capacity is the shifting of attention to different aspects of otherwise ambiguous stimuli to reliably earn predicted reward. Patients with frontal lobe damage or schizophrenia are well known to be poor at making such shifts in clinical tests such as the Wisconsin card sorting task. This type of attentional shifting is also impaired by ketamine in healthy human volunteers [50]. Recently, it was shown that NMDA receptor blockade (using the non-competitive antagonist MK801 infused into the rat prefrontal cortex) impaired the capacity to shift attentional set, causing animals to perseverate (maladaptively repeat) in responding to visual cues when the rewards were instead predicted by the texture of the floor [51]. AMPA receptor blockade within the PFC impaired performance in a nonspecific way, affecting not only shifting but also discrimination learning, which is reminiscent of its generalized effects on memory processes.

These exciting data indicate that some of the highest levels of cognitive functioning, impaired in such complex conditions as schizophrenia, are also susceptible to NMDA receptor manipulation, and therefore impairments might be remediated by glutamate receptor agents. Whether such basic processes as LTP will be shown to underlie such rapid flexibility in behavioural control, or whether such effects depend on another pharmacological action at NMDA receptors, is not yet clear. However, results such as these suggest that a much more sophisticated understanding of what is meant by the term ‘neuronal plasticity’ is required. Almost 20 years following the original implication of NMDA receptors in spatial learning, the role of these receptors has expanded to include many other forms of learning and memory. Furthermore, distinct roles have emerged for unique glutamate receptor subtypes, with further understanding of more recently discovered subtypes on the horizon (Table 1). These developments have strong potential therapeutic implications, and it seems that the next decade of collaborative enterprise between behavioural pharmacology and neuroscience will probably uncover some of these promising possibilities.

## Figures and Tables

**Table 1:** Involvement of NMDA and AMPA receptors in cognitive functionsa,b

	**Visual recognition**		**Spatial learning**		
**Declarative memory**	NMDA (−)	AMPA (−)	NMDA (−)	AMPA (−)	
Encoding	−	↓	−	↓	
Consolidation	↓	↓	↓	↓	
Retrieval	−	↓	−	↓	

	**Appetitive and aversive conditioning**		**Extinction**		
**Associative conditioning**	NMDA (−)	AMPA (−)	NMDA (−)	NMDA (+)	AMPA (−)
Acquisition (encoding/consolidation)	↓	−	↓	↑	−
Performance (retrieval)	−	↓^c^			

**Executive functions**	NMDA (−)	AMPA (−)	AMPA (KO)		
Working memory	↓	↓^d^	↓		
Discrimination learning	↓	X^e^			

a Symbols: (−), pharmacological antagonism; (+), direct or indirect agonism; (KO), genetic knockout model; ↑, function is improved; ↓, function is impaired; −, function is not affected.

b Some effects are mediated in specific brain areas; see main text for details.

c Discriminated approach to appropriate stimulus disrupted; a generalized deficit.

d Low systemic doses impair performance only at long retention intervals.

e Generalized cognitive disruption.

## Chemical names

**HA966:**(+)-3-amino-1-hydroxypyrrolid-2-one
**LY326325:**(3*RS*,4a*RS*,6*RS*,8a*RS*)-6-[2-(1(2)*H*-tetrazole-5-yl)ethyl]decahydro-isoquinoline-3-carboxylic acid

## Glossary of psychological terms often used in behavioural pharmacology

**Conditioned stimulus (CS):**: generally a motivationally neutral stimulus such as light or auditory tone that gains salience by being predictive of an unconditioned stimulus or reinforcer such as food or electric shock.
**Consolidation:**: the memory process by which short-term memory representations (or ‘traces’) are made more permanent, leading to memory storage.
**Declarative memory:**: generally used to denote conscious recollection of autobiographical events (‘episodic memory’) or facts (e.g. routes or capitals of countries: ‘semantic memory’.
**Discriminated approach:**: a form of conditioning in which the animal is trained to approach one stimulus rather than another stimulus.
**Extinction:**: when the reinforcer (e.g. food or shock) associated with learning a particular task or response is omitted or withdrawn.
**Instrumental learning:**: a form of learning in which a voluntary action produces an outcome or goal (e.g. leading to a reward or the postponement of an aversive stimulus; both of these are termed reinforcers, hence the term reinforcement learning. This type of learning contrasts with Pavlovian (classical) conditioning.
**Learned contextual fear:**: a form of Pavlovian conditioning in which fear is conditioned to an entire environment or setting rather than a discrete CS.
**One trial ‘place memory’ test:**: a specialised test of spatial memory in which a specific location is associated with reinforcement (e.g. food or safety) in one trial only.
**Recognition memory:**: a simple form of declarative memory in which, for example, a visual object such as a face becomes familiar.
**Retrieval:**: the memory process by which memories are summoned from long-term storage, and converted from a ‘passive’ to an ‘active’ form.
**Stimulus–response habits:**: generally highly over-trained instrumental learning that is less dependent on the outcome or goal and is performed somewhat automatically, compared with normal levels of training.
**Unconditioned stimulus (US):**: generally a reinforcer such as food or shock that elicits unconditioned responses (such as salivation to food) and is subject to Pavlovian conditioning following several pairings with a predictive CS.
**Wisconsin card sorting task:**: a cognitive test in which the ability to deduce rules and shift rules on the basis of trial-and-error learning is required. It is often used clinically as a test of cognitive flexibility; patients with frontal lobe damage or dysfunction often fail by perseverating with the original rule.
**Working memory:**: not simply short-term memory, but memory that involves the manipulation (or ‘encoding’) of sensory representations into more-persistent representations during short periods of time. It is often equated with the ‘online’ maintenance of representations in the solution of a problem or the retrieval of memory.
